# Inhibition of U-87 MG glioblastoma by AN-152 (AEZS-108), a targeted cytotoxic analog of luteinizing hormone-releasing hormone

**DOI:** 10.18632/oncotarget.917

**Published:** 2013-03-07

**Authors:** Miklos Jaszberenyi, Andrew V. Schally, Norman L. Block, Mehrdad Nadji, Irving Vidaurre, Luca Szalontay, Ferenc G. Rick

**Affiliations:** ^1^ Veterans Affairs Medical Center, Miami, FL; ^2^ South Florida VA Foundation for Research and Education, Miami, FL 33125; ^3^ Department of Pathology, University of Miami, Miller School of Medicine, Miami, FL; ^4^ Division of Hematology/Oncology, University of Miami, Miller School of Medicine, Miami, FL; ^5^ Division of Endocrinology, Department of Medicine, University of Miami, Miller School of Medicine, Miami, FL

**Keywords:** glioblastoma multiforme, U-87 MG, cytotoxic, LHRHor GnRH receptors, AN-152, AEZS-108, nude mice, targeted therapy

## Abstract

Glioblastoma multiforme is the most frequent tumor of the central nervous system in adults and has a dismal clinical outcome, which necessitates the development of new therapeutic approaches. We investigated in vivo the action of the targeted cytotoxic analog of luteinizing hormone releasing hormone, AN-152 (AEZS-108) in nude mice (Ncr nu/nu strain) bearing xenotransplanted U-87 MG glioblastoma tumors. We evaluated in vitro the expression of LHRH receptors, proliferation, apoptosis and the release of oncogenic and tumor suppressor cytokines. Clinical and U-87 MG samples of glioblastoma tumors expressed LHRH receptors. Treatment of nude mice with AN-152, once a week at an intravenous dose of 413 nmol/20g, for six weeks resulted in 76 % reduction in tumor growth. AN-152 nearly completely abolished tumor progression and elicited remarkable apoptosis in vitro. Genomic (RT-PCR) and proteomic (ELISA, Western blot) studies revealed that AN-152 activated apoptosis, as reflected by the changes in p53 and its regulators and substrates, inhibited cell growth, and elicited changes in intermediary filament pattern. AN-152 similarly reestablished contact regulation as demonstrated by expression of adhesion molecules and inhibited vascularization, as reflected by the transcription of angiogenic factors. Our findings suggest that targeted cytotoxic analog AN-152 (AEZS-108) should be considered for a treatment of glioblastomas.

## INTRODUCTION

Malignant tumors are frequently difficult to treat using conventional chemotherapy treatment. However, targeted cytotoxic peptide analogs could overcome this problem. [1, 2] Since various neuropeptides play a pivotal role in carcinogenesis, their appropriate receptors can be targeted with cytotoxic peptide complexes.[1, 3] Targeting increases efficacy, while reducing toxic side effects[1] on innocent bystander tissues, because, through receptor internalization, the cytotoxic compounds selectively cross the cell membrane of the target cells.[1, 2, 4, 5] Our group has synthesized analogs of luteinizing hormone releasing hormone (LHRH), somatostatin, and bombesin linked to doxorubicin (DOX) or 2-pyrrolinodoxorubicin.[1-3, 6-8]

LHRH[9] and its receptor (LHRH-R) are not confined to the hypothalamic-pituitary axis.[10] In the periphery, the LHRH system coordinates gonadal functions and serves as a growth factor of benign conditions [11-13] and various malignancies.[10, 14] In the central nervous system (CNS), hypothalamic and extra-hypothalamic cell populations can be detected [15, 16], where dense immunostaining for LHRH and LHRH-R can be demonstrated.[15-17] Beside endocrine functions, these cells are involved in the regulation of the olfactory system, feeding, reproductive behavior and circadian rhythms [15, 16, 18]. The LHRH-positive subventricular zone, a frequent starting locus of primary glioblastoma multiforme (GBM) [19], often shows hypertrophy and hyperplasia, in the absence of steroid feed-back, in postmenopausal and andropausal subjects [17]. Since this age-group has the highest prevalence of GBM [20], and GBM tumors frequently show high expression of LHRH-R [21, 22], these findings suggest a regulatory role of the LHRH system in the evolution of brain cancer. The modulation of the LHRH system is used for the treatment of several cancers. LHRH agonists are the mainstay in the therapy of prostate cancer and act through the down-regulation of LHRH-R.[23] Moreover, our previous studies showed that the LHRH antagonist, cetrorelix[24], and the cytotoxic analog, AN-152[25] can be successfully used for the treatment of cancers of the reproductive system[25] and of other organs.[1, 2, 26-28]

In the present study, we first demonstrated LHRH-R expression on clinical samples of GBM and U-87 MG cells by immunohistochemistry (IHC) and Western blot. In vitro, U-87 MG cells were exposed to AN-152 and viable cells were determined by proliferation assay. Subsequently, the effectiveness of the cytotoxic analog, AN-152 was evaluated, in vivo, on the growth of U-87 MG tumors xenotransplanted into nude mice. To evaluate its mechanism of action, the most frequently involved “cancer pathway” genes were screened with real-time PCR arrays. Also, apoptotic processes and drug resistance were detected by specific kits. The ability of a chemotherapeutic drug to pass through the cell membrane and to accumulate within the cellular compartments of the neoplastic tissue is one of its most important pharmacodynamic features. Intracellular accumulation was tested by competition with a fluorescent test compound on multidrug resistance (MDR) pumps, by which cells get rid of toxic foreign molecules. Proteomic verification of functional and genomic changes was performed by Western blots and ELISAs.

## RESULTS

### LHRH-R expression

**Figure 1:**
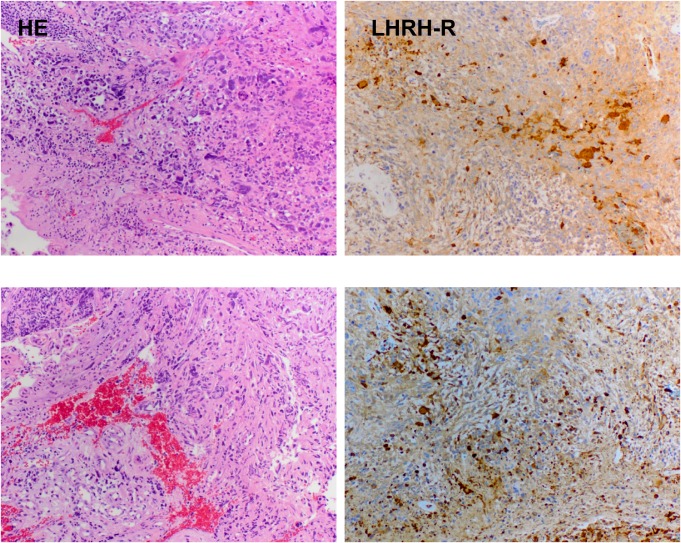
Expression of receptors for LHRH in two representative human GBM specimens The samples were stained by hematoxylin-eosin (panel HE) and IHC (with affinity-purified goat polyclonal antibody, Santa Cruz, panel LHRH-R). Magnification is 50×.

The dense expression of LHRH-R on clinical samples of GBM was shown by the positive reaction in the form of brown granules (Fig. 1).

### Animal studies

**Figure 2:**
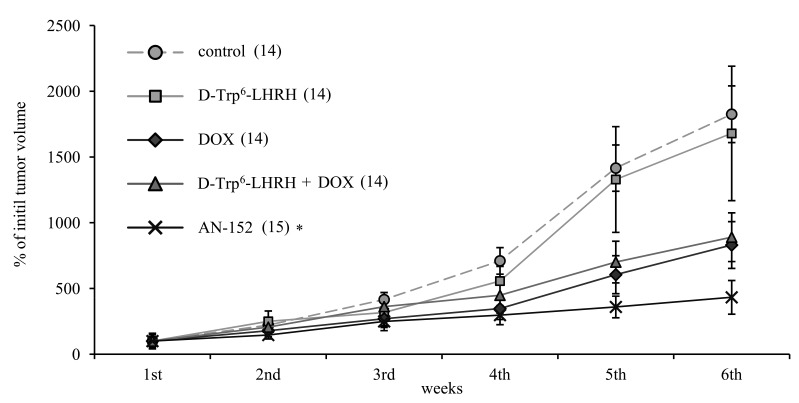
The effect of the cytotoxic LHRH analog, AN-152 (AEZS-108), on the growth of xenotransplanted U-87 MG, human glioblastoma tumors The pooled standard errors of the groups: control: 313.9; D-Trp^6^-LHRH: 645.3; doxorubicin (DOX): 267.8, D-Trp^6^-LHRH + DOX: 308.9; AN-152: 172.1. Numbers in brackets are the number of successfully grafted tumors. Numbers at the end of each line represents the tumor doubling times. * = p < 0.05 vs. control for the repeated measure evaluation of tumor growth progression curves.

Groups of nude mice bearing U-87 MG tumors were treated once a week for 6 weeks with AN-152, DOX, D-Trp6-LHRH mimicking the carrier molecule, or with the combination of DOX and D-Trp6-LHRH. Treatment with AN-152 produced the greatest inhibition (Fig. 2) and repeated measure ANOVA revealed significant effects (Within-Subject F5,62=17.87, p<0.01, Between-Subject F4,66=78.6, p<0.01). With pair-wise comparison only the effect of AN-152 was significant compared to the control (Tukey's HSD test: p<0.01 vs. control), which suggests that targeting greatly increased the effectiveness of therapy. A similar tendency could be observed in the length of tumor doubling times (given in days; control: 10.02, DOX: 13.75, D-Trp6-LHRH: 13.32, DOX + D-Trp6-LHRH: 10.32, AN-152: 19.88), although this effect did not prove to be statistically significant.

### Proliferation, Apoptosis and MDR assays

**Figure 3:**
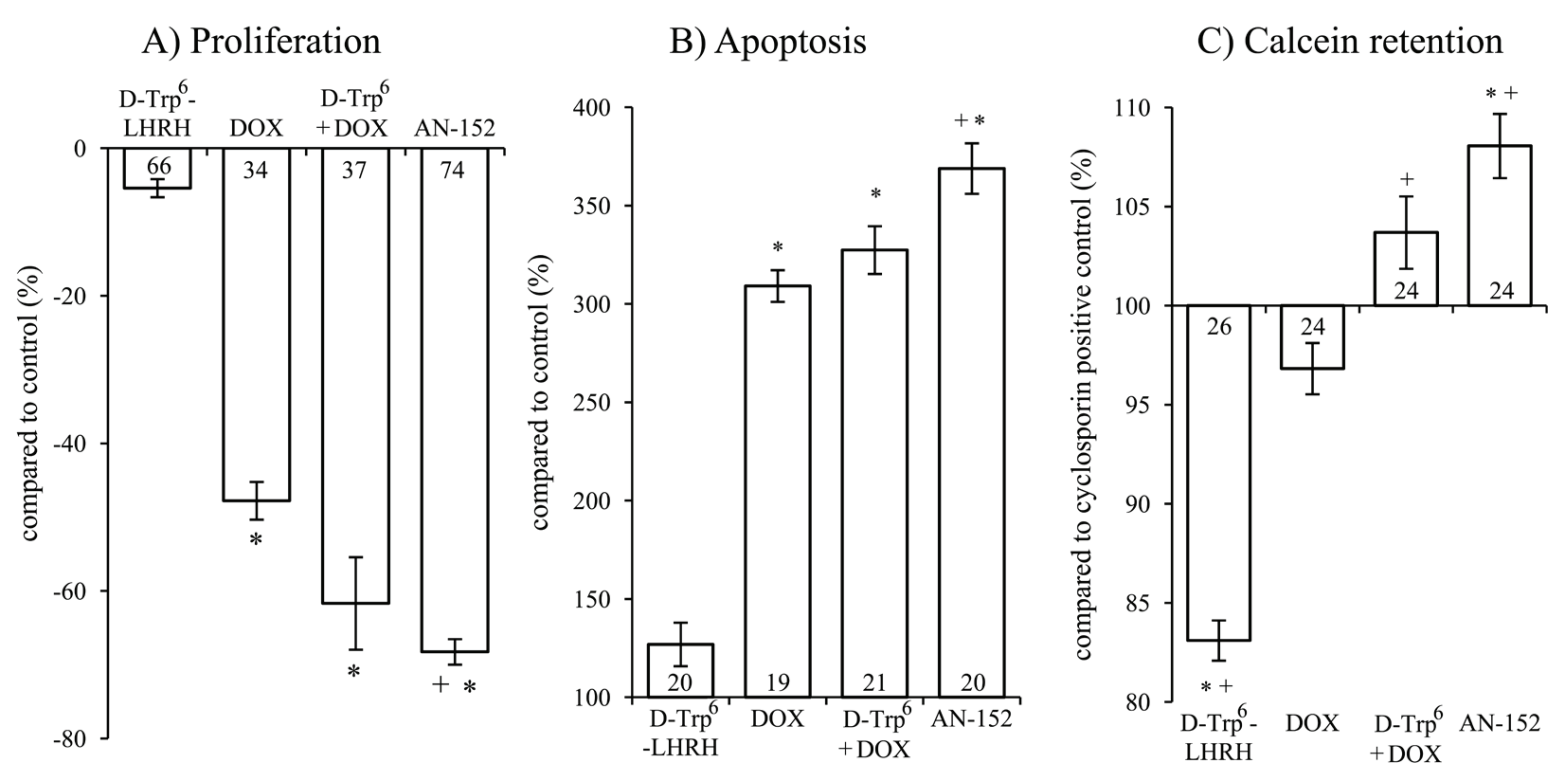
The effect of the treatment with AN-152 (AEZS-108) on the proliferation (A), apoptosis (B) and calcein retention (C) of U-87 MG cells Sample numbers at the bottom of each column refer to the seeded wells, which underwent the given treatment. Abbreviations: DOX: doxorubicin, D-Trp^6^ + DOX: D-Trp^6^-LHRH + doxorubicin. * = p < 0.05 vs. control (n=24); + = p < 0.05 vs. DOX.

Single exposure to AN-152 brought about an almost 70 % inhibition of tumor cell growth (Fig. 3/A, F4,284=374.49, p<0.01; Tukey's post hoc p<0.01 vs. control in both cases). Treatment with DOX or the unconjugated combination also led to growth suppression but to a lesser extent. The group treated with AN-152 was statistically different from the DOX treated one (Tukey's post hoc: p<0.01 vs. DOX).

Both DOX and AN-152 elicited a significant increase in apoptosis (F4,91=110.61, p<0.01, Tukey's post hoc test: p<0.01 vs. control). Again, AN-152 was the most effective (with almost 250 % increase) and its effect was statistically more significant than that of DOX (Tukey's post hoc: p<0.01 vs. DOX; Fig. 3/B). These assays, measuring viable cell count and cell death, confirmed our in vivo findings. Further, in both cases, the targeted cytotoxic compound was significantly more effective than DOX itself.

In the MDR assay, only AN-152 caused a greater increase in calcein retention than the Cyclosporin-A positive control (F4,117=46.8, p<0.01; Tukey's post hoc test: p<0.01 vs. positive control). Treatment with AN-152 proved to be significant even compared to DOX (Tukey's post hoc test: p<0.01 vs. DOX; Fig. 3/C). Presumably receptor mediated internalization leads to significantly higher intracellular concentrations of DOX, which, in turn leads to overload of MDR transporters that try to eliminate toxic, foreign compounds from the cell. The increased competition on the MDR transporters, eventually results in increased calcein retention.

### PCR experiments

PCR array studies revealed a significant antitumor effect of AN-152 on the marker regulators of cell proliferation and cell death (nuclear factor κB (NF-κB), platelet derived growth factor (PDGF), metastasis associated 1 family, member 2 (MTA2)), contact and humoral control (integrins, tumor necrosis factor receptor 10β (TNF-R10β)), invasion (MMP-9, urokinase plasminogen activator (uPA)) and metastasis formation (melanoma cell adhesion molecule (MCAM), MTA2). Compared to the parallel treatments AN-152 generally elicited more profound changes in gene expression and its activity on invasion, angiogenesis and metastasis markers (uPA, MMP-9, MCAM, MTA2) was especially significant (Table 1).

**Table 1:** Relative expression of genes related to tumor growth

Gene	D-Trp6-LHRH	DOX	D-Trp6 + DOX	AN-152
Angiopoietin 1	0.71	0.51	0.65	0.2
Insulin-like growth factor 1	0.33	0.5	0.22	0.08
Integrin, α4 (α4 subunit of VCAM-1 receptor)	0.25	0.33	0.59	0.46*
Integrin, αV (domain of vitronectin receptors)	0.25*	0.38	0.57	0.32*
Integrin, β5 (domain of vitronectin receptor)	1.14	1.3	0.84	0.46*
Mitogen-activated protein kinase 1	0.65*	1	0.19	0.22
Melanoma cell adhesion molecule	0.24*	0.57	0.19	0.06*
Matrix metalloproteinase 9 (gelatinase B)	0.34	0.73	0.5	0.2*
Metastasis associated 1 family, member 2	0.6	1.02	1.2	0.68*
Nuclear factor κB	0.85	1.13	0.11*	0.6*
Phosphoinositide-3-kinase, regulatory subunit 1α	0.49*	0.74	0.67	0.67*
Plasminogen activator (urokinase)	1.34	1.79*	0.8	0.6*
Platelet-derived growth factor α	0.83	1.52	4.09	0.36*
Serpin, clade B, member 5 (maspin)	0.16	0.14	17.67*	4.27
Tumor necrosis factor receptor superfamily, 10β	0.84	1.63	1.14	1.87*
V-raf-1 murine leukemia viral oncogene homolog 1	0.74	1.06	1.13	0.63*

*In vivo* glioblastoma specimens were evaluated by Cancer Pathway RT2 Profiler PCR Array system. Only genes with at least three-fold or statistically significant changes are represented. Four tumor samples from each group were analyzed. Relative expressions are compared to the control. *p < 0.05 vs. control. Abbreviations: DOX: doxorubicin, D-Trp6 + DOX: D-Trp6-LHRH + doxorubicin, VCAM: vascular cell adhesion molecule.

### Western blot and ELISA experiments

**Figure 4:**
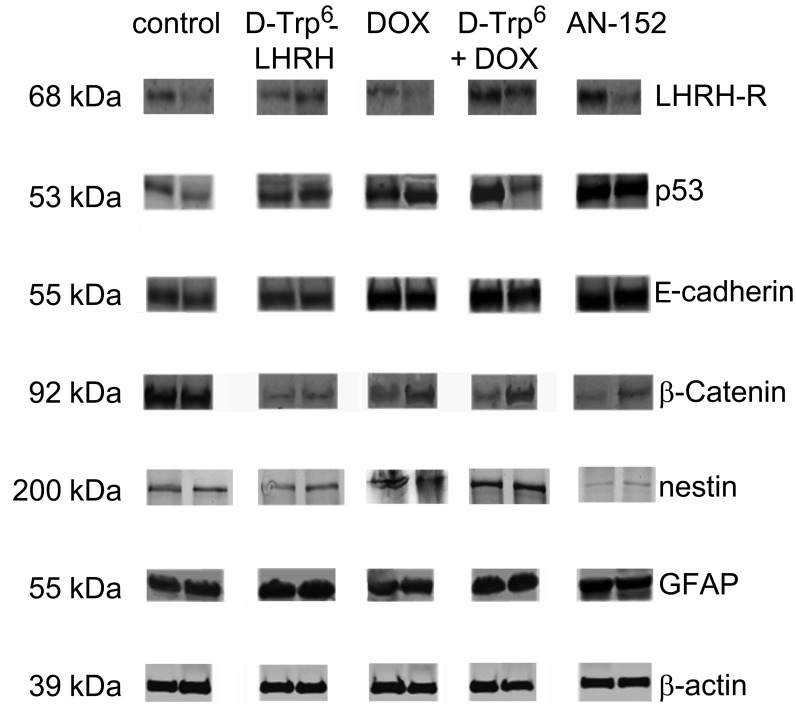
Western blot analyses of LHRH receptor and tumor marker expression following AN-152 treatment Abbreviations: LHRH-R: luteinizing hormone releasing hormone receptor, GFAP: glial fibrillary acid protein, DOX: doxorubicin, D-Trp^6^ + DOX: D-Trp^6^-LHRH + doxorubicin. * = p < 0.05 vs. control.

Western blot studies (Fig. 4) verified the expression of LHRH-R expression in U-87 MG xenograft samples. Importantly, AN-152 treatment did not induce any down-regulation of LHRH-R. These experiments also verified the remarkable up-regulation of the tumor suppressor and pro-apoptotic p53 by the cytotoxic analog (IDVs: control: 4102.7±1096.7, AN-152: 14895.0±1153.4). Further, AN-152 reestablished contact inhibition through up-regulation of E-cadherin (IDVs: control: 6636.6±2042.0, AN-152: 21925.7±163.7) and down-regulation of β-catenin (IDVs: control: 16392.5±2155.3, AN-152: 1349.6±757.2). One of the most important results of the Western blot studies was that the cytotoxic analog inhibited the expression of the primordial, neuroectodermal stem cell marker, nestin (IDVs: control: 3149.1±157.1, AN-152: 739.5±143.5) and stimulated the synthesis of the maturation antigen, GFAP (IDVs: control: 12007.7±2209.8, AN-152: 16179.6±758.845).

**Figure 5:**
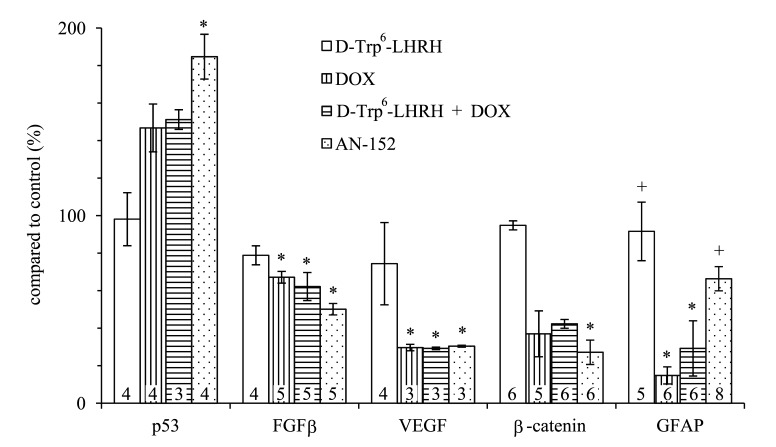
The effect of the treatment with AN-152 (AEZS-108) on the release of cytokines and signal transducers verified by ELISA experiments Sample numbers at the bottom of each column refer to the seeded wells, which underwent the given treatment. Abbreviations: GFAP: glial fibrillary acid protein, DOX: doxorubicin. * = p < 0.05 vs. control; + = p < 0.05 vs. DOX.

The level of several oncogenic cytokines and tumor suppressor molecules was modified by the AN-152 treatment as shown by ELISA (Fig. 5). First, these studies, using homogenized cell culture samples, confirmed the increase of p53 and the decrease of β-catenin observed in the Western blot experiments. The statistical analyses showed significant changes in both cases (F4,14=7.2, p<0.01, Tukey's post hoc test: p<0.01 vs. control (n=4) for p53 and F4,23=32.48, p<0.01, Tukey's post hoc test: p<0.01 vs. control (n=5) for β-catenin). In addition, FGFβ, one of the decisive markers of glial growth, and VEGF, the most important factor of tumor vascularization and nutrition, were significantly down-regulated by AN-152 treatment (F4,19=7.8, p<0.01, Tukey's post hoc test: p<0.01 vs. control (n=5) for FGFβ; F4,11=4.8, p<0.05, Tukey's post hoc test: p<0.05 vs. control (n=3) for VEGF). The expression of the maturation marker, GFAP, was reduced by the treatment with DOX (F4,26=14.67, p<0.01, Tukey's post hoc test: p<0.01 vs. control (n=6)). This suggests dedifferentiation or the survival of an immature aggressive proliferation prone phenotype. AN-152 treatment caused a much lesser reduction (p<0.01 vs. DOX), which suggests that the cytotoxic analog, most importantly, may inhibit the survival of resistant stem cell-like clones.

## DISCUSSION

Our IHC and Western blot analyses clearly demonstrated that LHRH-R is expressed on human GBM cells, suggesting a role of intrinsic LHRH secretion in the autocrine/paracrine control of GBM cells (Fig. 1, 4). The treatment with ligands of LHRH-R did not down-regulate the LHRH receptors, which augurs well for continuing long term therapy with AN-152. D-Trp6-LHRH representing the carrier molecule exerted only a weak effect on in vivo and in vitro tumor growth (Fig. 2 Fig. 3/A) and the expression of adhesion molecules and growth factors (Table 1, Fig. 4-5). However, in the case of some oncoproteins such as MAPK1, the MAPK activator, V-raf-1 murine leukemia viral oncogene homolog 1 (Raf-1), phosphoinositide-3-kinase (PI3K) and the differentiation marker, GFAP, the changes reached the level elicited by the AN-152 treatment.

AN-152 elicited profound inhibition of tumor growth both in vivo (Fig 2) and in vitro (Fig. 3/A), and the effect of the cytotoxic analog exceeded that of DOX (Fig. 2-5). This intensified antitumor potential appears to be related to the “homing property” of the cytotoxic analog[4], which can lead to an increased competition on the MDR transporter proteins reflected by the increased calcein retention in the MDR studies. However, according to the PCR experiments, D-Trp6-LHRH mimicking the carrier molecule, may also act as an inhibitor of the MAPK pathway (Table 1). This may lead to a direct inhibition of MDR-1 protein translocation/activation[29] and decreased resistance, which confirms our previous findings.[4, 30]

According to the signal transduction studies, AN-152 influenced the cell-cycle, differentiation, contact and humoral control, angiogenesis and invasion. The cytotoxic analog increased levels of the pro-apoptotic tumor suppressor, p53, which molecule is frequently mutated in GBMs[31, 32] (Fig. 4-5). The increase of p53 can be related to the down-regulation of MTA2, which plays a role in the deacetylation and breakdown of p53.[33] The apoptosis stimulating potential of AN-152 is supported by the increased expression of the death domain containing TNF-R10β (Table 1).[34] The cytotoxic analog also suppressed the antiapoptotic NF-κB expression.[35]

AN-152 mitigated the release of several glial growth factors, such as insulin-like growth factor I (IGF-I), FGF, and PDGF and also blocked the interwoven MAPK and PI3K-protein kinase B (PKB)/Akt survival pathways (Fig. 5, and Table 1).[36] The cytotoxic analog decreased the expression of MAPK-1, Raf-1 and the regulator subunit 1 (PIK3R1) of PI3K. Since growth promoting cytokines[37] are able to stimulate the survival cascades[38], both direct and indirect inhibition can explain the antiproliferative activity of AN-152.

The treatment with AN-152, according to the proteomic studies, also influenced the expression of the glial differentiation markers, nestin and GFAP (Fig. 4-5).[39, 40] During astrocyte maturation, nestin as a common neuroectodermal marker progressively disappears, while GFAP becomes the characteristic intermediary filament. In our experiments, DOX treatment elicited an increase in the expression of nestin and a decrease in the transcription of GFAP suggesting that, an undifferentiated, pluripotent clone became dominant upon treatment. Such cancer stem-like cell populations may retain the capability of expressing a wide array of resistance factors, which later would normally disappear, through differentiation.[41] Concersely, the cytotoxic analog elicited opposite changes, perhaps due to its “homing” neuropeptide molecule (Fig. 4-5).

AN-152 also exerted a beneficial effect on contact and humoral regulatory factors, which restrain tumor growth at a population level (Fig. 4-5, and Table). The increase in E-cadherin expression, and β-catenin degradation (Fig. 4-5) may inhibit the so-called cadherin-switch, when the expression pattern from E-cadherin changes to N-cadherin, resulting in loss of adhesion and stimulation of invasion.[42, 43] Moreover, AN-152 decreased the transcription of three (α4, αV, β5) contact activator integrin domains (Table 1), which are frequently over-expressed in GBM tumors.[44]

Beside the pronounced inhibition of VEGF secretion (Fig. 5), the cytotoxic analog decreased the expression of angiopotein-1[45], and MCAM (Table 1), which are beneficial effects, since the suppression of angiogenesis is one of the most important complementary therapeutic approaches in the case of GBMs.[46] Beside neovascularisation, MCAM regulates cell proliferation through the PKB/Akt pathway, stimulates cell migration, increases the expression of MAPK and the proteolytic MMPs.[47] AN-152 also suppressed the expression of MMP-9 and the uPA and augmented the local tumor suppressor, maspin, level thus inhibiting the invasive capability activity of tumor cells (Table 1).[48, 49]

The present results, taken together with our previous findings promote the concept of a new, multi-faceted chemotherapeutic paradigm in the treatment of GBM, based on targeted peptide analogs. This concept is enhanced by the fact that analogs of hypothalamic peptide-hormones can cross the blood brain barrier.[50] Our results show that AN-152 is a versatile multi-pronged hybrid molecule which, beside direct antiproliferative and pro-apoptotic activity, elicits maturation. These features establish AN-152 as a very promising therapeutic option against brain cancers which express LHRH-R. In view of ongoing encouraging clinical phase I//II/III trials with AN-152 (also denoted by its commercial designation, AEZS-108) in gynecological, prostatic and urothelial cancers[1, 3, 26, 28], our findings strongly suggest a significant step forward in the successful therapy of malignant gliomas, curative treatment of which is not yet available.

## METHODS

### Ethics Statement

Investigation has been conducted in accordance with the ethical standards and according to the Declaration of Helsinki and according to national and international guidelines and has been approved by the authors' institutional review board.

### Peptides and chemicals

The cytotoxic LHRH conjugate, AN-152, and the LHRH agonist D-Trp6-LHRH were synthesized in our laboratory as described[51]. DOX hydrochloride was obtained from Chemex Export-Import Gmbh (Vienna, Austria). The compounds were dissolved for injection in 5% (w/v) aqueous D-mannitol solution as vehicle.

### Animal experiments

Six-week-old female nude mice (Ncr nu/nu) were obtained from the NCI (Bethesda, MD). The animals were maintained according to the guidelines of the Institutional Animal Care and Use Committee, as described previously.[52, 53]

### *In vivo* study design

The animal studies with the U-87 MG GBM cell line (American Type Culture Collection, Manassas, VA) were performed as described previously [52]. Donor mice were injected in the flanks with 1×106 glioblastoma U-87 MG cells. After 4 weeks, tumor tissue grown in these donor animals was minced and passed through a wire mesh and a 150 μl suspension of this was injected s.c. into experimental nude mice. The experiment was initiated when U-87 MG tumors had reached a volume of approximately 70 mm3. Mice bearing xenografts were randomized into 5 groups of 10 mice each with a random number generator function of Microsoft Excel (Microsoft Corp. Redmond, WA). The groups received, once weekly, the following intravenous treatment for 6 weeks, respectively: group 1: (control, 14 tumors, 100 μl vehicle solution); group 2: (DOX, 14 tumors); group 3: (D-Trp6-LHRH, 14 tumors); group 4: (DOX + D-Trp6-LHRH, 14 tumors); group 5: (AN-152, 15 tumors). The concentration of the compounds was equimolar (413 nmol/20g) and was adjusted to the maximum tolerable dose of the cytotoxic drugs, which had proved the most effective in our previous oncological studies.[5-7, 52] Tumor dimensions were measured with microcalipers once a week and volume was calculated using the formula: (length × width × height × π)/6. Tumor doubling time was calculated using the formula: (study duration × LOG 2)/(LOG final tumor volume - LOG initial tumor volume).

### *In vitro* experiments

### IHC

Formalin-fixed, paraffin-embedded, surgically removed tissue samples were used for IHC. Three micron paraffin sections were stained by hematoxylin and eosin to confirm the presence of GBM. Adjacent serial sections were utilized for immunoperoxidase staining following standard protocols as previously described[54] and using a polyclonal antibody to LHRH-R (GnRHR-N20, Santa Cruz Biotechnology, Santa Cruz, CA). The sections were then counterstained with hematoxylin. Human pituitary glands (anterior lobe) obtained from autopsy were used as positive controls. The use of archival samples of GBM was approved by the institutional review board.

### Cell culturing

U-87 MG cells were cultured in EMEM (ATCC) medium (supplemented with 10% FBS, (ATCC) and 0.1% penicillin/streptomycin) at 37°C and 5% CO2 atmosphere. This cell line is classified as grade IV GBM and was characterized and deposited by J. Ponten and associates.[55] As a treatment, those doses (100 nM) of AN-152 were used which had proved most effective in our previous studies[56]. Other compounds were administered in equimolar concentrations.

### Proliferation and apoptosis assays *in vitro*

For proliferation studies, 104 cells/well were seeded in 100 μl medium, in a 96-well plate and were then incubated for 24 h in a humidified incubator at 37 °C. Next, culture medium was replaced with FBS free medium (starvation) for 24 h. After another 24 h, the cells were exposed to complete medium containing 100 nM of AN-152, DOX, D-Trp6-LHRH, or the combination of DOX and D-Trp6-LHRH. The cells were then incubated for 48 h. The effect of the compounds on cell proliferation was evaluated by using the 3-(4,5-Dimethylthiazol-2-yl)-2,5-diphenyltetrazolium bromide (MTT) assay (CellTiter 96® Non-Radioactive Cell Proliferation Assay, Promega, Madison, WI, USA), according to the manufacturer's instructions with the help of a Victor3 multilabel counter (Perkin-Elmer, Waltham, MD, USA)(5). Determination of apoptosis was performed on freshly seeded cell samples (104 cells/well, in 100 μl media, in a 96-well plate) by the Multi-Parameter Apoptosis Assay Kit (Cayman Chemical Company, Ann Arbor, MI), according to the manufacturer's instruction.

### Multidrug resistance assay

This assay was performed according to the manufacturer's instructions (Cayman Chemical Company, Ann Arbor, MI). U-87 MG cells were seeded in 5×104 cells/well density in 100 μl medium, to 96-well, black, clear bottom plates and were grown overnight in a humidified incubator at 37 °C. The next day the medium was discarded, and the cells were treated with medium containing 100 nM of AN-152, DOX, D-Trp6-LHRH, or the combination of DOX and D-Trp6-LHRH. As a positive control Cyclosporin-A solution was used in 1/1000 dilution according to the manufacturer's description. Afterwards, the cells were incubated for 1 h, then calcein AM/Hoechst dye combined staining solution was added. Fifteen minutes later both cell density (at excitation and emission wavelengths of 355 nm and 465 nm, respectively) and calcein retention (at excitation and emission wavelengths of 485 nm and 535 nm, respectively) were detected with the help of a Victor3 multilabel counter (Perkin-Elmer, Waltham, MD, USA). Relative calcein retention values were expressed as a function of cell density.

### Total RNA isolation and reverse transcription

Total RNA was isolated from representative, DNAse treated, U-87 MG tumor samples using a NucleoSpin kit according to the manufacturer's instructions (Macherey-Nagel Inc., Bethlehem, PA). Four tumor samples from each group were analyzed. The yield and the quality of RNA samples were determined spectrophotometrically using 260 nm, and 260/280 and 260/230 nm ratios. The synthesis of cDNA was performed as described[57]. Briefly, 1 μg of RNA from each sample was reverse-transcribed into cDNA by RT First Strand kit (Qiagen). Reverse transcription was done in a Veriti 96-well thermal cycler (Applied Biosystems).

### Cancer Pathway Finder quantitative PCR array

The Human Cancer Pathway Finder quantitative PCR array (PAHS-033A, Qiagen) used in our study contains 84 unique genes related to cell proliferation, apoptosis, cell cycle, angiogenesis, invasion and metastasis. All PCR arrays were studied using iQ5 Multicolor Real-Time Detections System (Bio-Rad). All genes represented by the array showed a single peak on the melting curve characteristic of the specific products. Four tumor samples from each group were analyzed. Data analysis of gene expression was performed using Excel based PCR Array Data Analysis Software provided by the manufacturer (Qiagen): fold-changes in gene expression were calculated using the ΔΔCt method and five stably expressed housekeeping genes (B2M, HPRT1, RPL13A, GAPDH, and ACTB) were used for normalization of the results.

### Western blot analyses

Proteins from the tumor tissue were isolated using the Macherey-Nagel NucleoSpin kit. The protein concentrations of the supernatant were determined by NanoDrop (NanoDrop Technologies Inc., Wilmington, DE). Equal amounts of protein were resuspended in sample loading buffer (0.25 M Trizma Base, 8% SDS, 40% glycerol, 0.004% bromophenol blue, 4% b-mercaptoethanol; pH 6.8), boiled for 3 min and separated by 12% SDS-polyacrylamide gel electrophoresis. Proteins from the gel were transferred onto nitrocellulose membranes, which were blocked with 50-50% Tris-buffered saline (TBS) (20 mM Tris-HCl pH:7.5, 150 mM NaCl): Odyssey blocking buffer for 1 h at room temperature, followed by an overnight incubation at 4°C with the following primary antibodies: nestin (cat. no.: ab92391), glial fibrillary acid protein (GFAP) (cat. no.: ab48050, both from AbCam Inc., Cambridge, MA), LHRH-R (sc-13944), β-actin (cat. no.: sc-47778, both from Santa Cruz Biotechnology, Inc., Santa Cruz, CA), β-catenin (cat. no.: 9562), E-cadherin, (cat. no.: 3195), p21 (cat. no.: 2947), p53 (cat. no.: 9282, all from Cell Signaling Technology Inc. (Danvers, MA). The signals were developed by incubating the membrane for 1 h at room temperature with the appropriate Infrared IRDye-labeled secondary antibodies (1:10000; LI-COR Biosciences, Lincoln, NE) and were then visualized with the Odyssey Infrared Imaging System (LI-COR Biosciences, Lincoln, NE). The protein bands were quantified using V3.0 software (LI-COR Biosciences, Lincoln, NE) and integrated densitiy values (IDV)s of duplicate experiments were calculated.

### ELISA assays for the determination of oncoprotein and tumor suppressor expression

U-87 MG cells (105 cells per well) were seeded onto 6-well plates, cultured overnight, and then exposed to the previously outlined treatments (100 nM of DOX, D-Trp6-LHRH, AN-152 or the combination of DOX and D-Trp6-LHRH) for 24 hours. Concentrations of specific proteins in cell lysates were determined according to the manufacturer's instructions. Human p53 and vascular endothelial growth factor (VEGF) ELISA kits were purchased from Biovendor, LLC (Candler, NC), fibroblast growth factor (FGF) basic human ELISA kit was obtained from AbCam Inc., total β-Catenin ELISA kits were received from Cell Signaling Technology, and GFAP human ELISA kit was purchased from Cayman Chemical Company. One or two plates were run and readings were normalized to protein concentrations determined by NanoDrop (NanoDrop Technologies Inc., Wilmington, DE).

### Statistical analyses

Statistical analyses were performed using either t-test for independent samples (two-sided, for PCR assays), univariate analysis of variance (ANOVA) (in vitro studies) or repeated measure ANOVA (in vivo experiment). ANOVA was followed by Tukey's post hoc tests for group-wise comparisons. Results are expressed either as the means ± SEM (in vitro studies) or as means and pooled standard errors (in vivo studies). Differences with p<0.05, compared to the control, were considered statistically significant. Data reductions and statistical analyses were performed by SigmaPlot 12.0 (Systat Software, Inc., Chicago, IL) and IBM SPSS Statistics 20.0 (IBM Corporation, Armonk, NY).
